# Performance of Green Concrete and Inorganic Coating Materials

**DOI:** 10.3390/ma14040832

**Published:** 2021-02-09

**Authors:** Sung-Ching Chen, Wei-Ting Lin, Ran Huang, Hui-Mi Hsu

**Affiliations:** 1 School of Civil and Architectural Engineering, East China University of Technology, Nanchang 330013, China; jaosn314@ecit.edu.cn; 2 Department of Civil Engineering, National Ilan University, No.1, Sec. 1, Shennong Rd., Ilan 26047, Taiwan; 3 Department of Harbor and River Engineering, National Taiwan Ocean University, Keelung 20224, Taiwan; ranhuang@mail.ntou.edu.tw; 4 Department of Materials Science and Engineering, National Dong Hwa University, Hualien 97401, Taiwan; hmhsu@niu.edu.tw

**Keywords:** inorganic coating material, green concrete

## Abstract

Green concrete (GC) was developed for realizing sustainable development, recycling waste materials, and reducing environmental pollution. For the practical use of GC, various harmful materials must blocked from entering its cracks and pores; and its strength and durability must be improved. The use of an inorganic coating material (ICM) for GC effectively prevents the intrusion of harmful materials and repairs the concrete. ICMs can reduce the permeability and increase the durability of concrete. This study investigated GC, construction waste, and ICMs and used recycled sand and gravel as well as construction waste as substitutes for cement. The results indicate that the coarse aggregate substitution, water-binder ratio, and recycled fine aggregate substitution must be controlled suitably in GC. Furthermore, the coating layer, fine aggregate substitution, and aging of the ICM mut be controlled suitably. GC with an ICM showed poorer performance than conventional concrete, mainly because of the high porosity. Nonetheless, the ICM somewhate reduces the porosity and resists the penetration of chloride ions, thereby promoting concrete quality.

## 1. Introduction

Reinforced concrete is the main material used in construction projects [1,2]. Cement is an important binding material for concrete. However, the cement industry has considerable energy consumption, and large amounts of carbon dioxide are emitted. Therefore, effectively reducing cement consumption will help reduce energy consumption and carbon dioxide emissions. Toward this end, cementitious materials with low energy consumption and low pollution must be developed to replace ordinary Portland cement. For realizing sustainable development, green materials have been incorporated into conventional concrete [3,4,5,6,7,8]. Green materials reduce the environmental impact and even improve the performance of concrete [9,10,11]. The use of recycled aggregates or waste in concrete can effectively transform waste into resources. Therefore, a robust recycling mechanism must be established for realizing sustainable development. Doing so will satisfy the basic requirements of construction projects in terms of economic benefits, environmental protection, and energy conservation; reduce environmental impact and damage; and realize sustainable development.

After the waste concrete was broken, sieved, and processed, the recycled aggregate, can be divided into recycled coarse aggregates and recycled fine aggregates. The influence of recycled coarse-grained materials on the properties of concrete was reported by previous studies [12,13,14,15,16,17,18,19,20], which show that recycled coarse-grained materials can replace natural coarse-grained materials to mix concrete. This kind of concrete has good properties, and can be used as structural materials. Many countries have also formulated relevant specifications for the use of recycled coarse-grained materials to manufacture concrete, which have been widely used in construction projects. Research on recycled fine-grained materials generally use recycled fine-grained materials of different proportions to replace natural fine-grained materials, and then make concrete for various tests to explore the influence of recycled fine-grained materials on concrete properties [21,22,23,24,25]. The results show that the fine recycled material with better quality can replace the fine natural material in a higher proportion of concrete.

The aim of the present study is to achieve the sustainable use of various waste materials and reduce environmental pollution caused by waste materials [26,27,28,29,30]. Green concrete (GC) is a kind of environmental protection material. Compared with ordinary concrete, it has better recycling and reduces the emission of harmful substances. It can not only slow down environmental pollution, but also maintain the balance of ecology and nature. It has important significance in energy saving, the engineering economy, and environment. The green definition aims to show that in order to save resources and not damage the ecological environment, it is more conducive to the sustainable development of the environment, it not only meet the needs of users, but also does not harm the ecology and environment; the cost of waste treatment is omitted. In GC, to prevent harmful substances from invading the surface layer and damaging the reinforcement, a suitable coating material must be used to provide a barrier effect to ensure the performance of concrete and prolong the service life of the structure [31,32].

Although various coating materials are available, most are harmful to the environment or the human body [33]. Further, with time, coatings can age and peel off, thus resulting in the loss of their concrete protection function. This study examined whether the combination of GC, industrial waste, and an inorganic coating material (ICM) can overcome this problem and achieve a sustainable protection function [34,35,36,37,38,39]. ICM is inorganic in nature and does not contain any organic chemical materials or solvents. In the production and application period, it is odorless, has acid resistant characteristics, is good for the environment and ecology, so it belongs to green materials.

There are many types of concrete surface treatments from different angles or requirements [40], including chemical composition—silane, siloxane, silicate, silicone, acrylic, epoxy, urethane, or cementation. In addition, functional properties such as beauty, blocking liquid penetration, or gas penetration. Additionally, the mechanism action, such as coating, pore hydrophobic treatment, pore plugging treatment, or coating. Further, the external environment such as chemical erosion environment, chlorpyrifos erosion environment, or humid environment [41]. Addittionally, the appearance such as powder, liquid, and coil, etc. ICM belongs to coating. The coating is mainly made of a thick layer of water material, which produces a physical additional barrier to prevent harmful substances from entering the concrete. The cement mortar can be modified by latex polymer to provide a dense impermeable barrier and enhance the adhesion to the substrate, or the cement-based crystalline coating material can be combined with chemical substances to provide pore plugging and produce a protective thick film on the outer layer [42]

Specifically, waste is used to replace a part of the cement, and concrete filling materials are replaced with recycled coarse and fine aggregates. Then, the protective effect of an ICM on the GC surface is discussed.

## 2. Materials and Methods

### 2.1. Materials

#### 2.1.1. Solar Photovoltaic Glass Powder

Solar photovoltaic glass powder provided by Zunhong Environmental Protection Co., Ltd. (Keelung, Taiwan) is made by self-breaking and grinding by ball mill. Table 1 lists the chemical composition analysis results of the solar photovoltaic glass powder used in this study; its main components were SiO_2_ (76%), Na_2_O (9%), and CaO (6%) by X-ray diffraction (XRD) spectral analysis provided by panalytical x’pert Pro MPD in Malvern Panalytical Ltd. (Malvern, UK). The compressive strengths of a mortar prepared with a 20% solar photovoltaic glass powder substitution for cement on a mass basis are compared to those of a control mortar according to ASTM C311 [43]. The results showed that the activity index was 88%, 80%, and 85% at 7, 28, and 56 days of age. The strength activity index was 75% higher than that of OPC (control group), which met the requirements of ASTM C618 [44]. In this study, solar photovoltaic glass powder was used to replace part of the cement in the production of concrete specimens.

#### 2.1.2. ICM

Inorganic coating material (ICM) came from XYPEX Chemical Corporation (Vancouver, BC, Canada). Table 2 lists the constitutents of the ICM used in this study. This material was a gray powder consisting of cement, silica sand, and other compounds [45]. The ICM was mixed with water to form a thick paste, and this paste was then evenly brushed on the substrate surface to form a ~1.5-mm-thick layer.

#### 2.1.3. Natural Coarse and Fine Aggregate

The fine-grained material is used for river sand from Lanyang River. The basic physical properties and gradation distribution of the fine-grained material are shown in Table 3 and Table 4.

**Figure 1:**
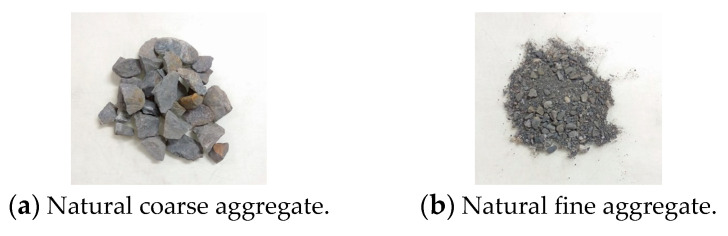
Appearance of natural coarse aggregate.

This study uses the coarse-grained materials produced in Lanyang River. The physical properties including specific gravity, water absorption, and maximum particle size are shown in Table 5 and Table 6. The appearance of natural coarse-grained and fine-grained materials is shown in Figure 1.

#### 2.1.4. Recycled Coarse and Fine Aggregate

The recycled fine aggregate used in this research institute is provided by Zunhong Environmental Protection Co., Ltd. (Keelung, Taiwan) and produced by the equipment of the Jilong sand and stone plant (Yilan, Taiwan). The design strength of the self-made concrete block in the sand and gravel plant is 280 kgf/cm^2^ because the sand and gravel plant can only deal with the natural river bed soil and stone and cannot use construction waste for processing. After curing for 56 days, it was disintegrated to make recycled pellets. The basic properties of the granular materials, including specific gravity, water absorption, maximum diameter, and fineness modulus, are shown in Table 7 and Table 8. The passing percentage of the granular materials conforms to ASTM C33 [47].

**Figure 2:**
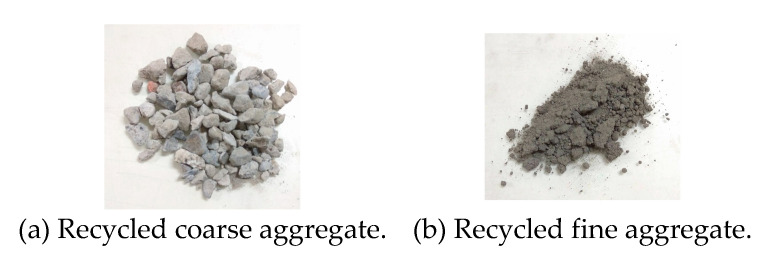
Appearance of recycled coarse aggregate.

**Figure 3:**
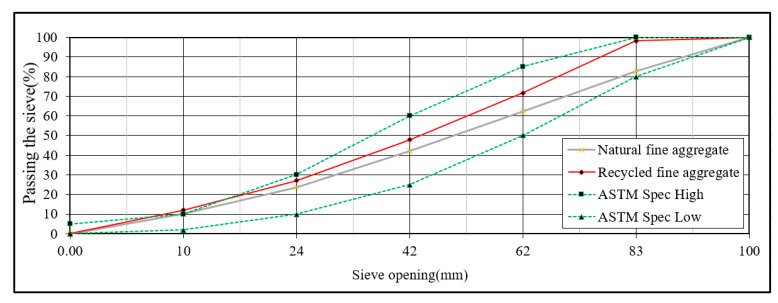
Particle size distribution curve of natural and recycled fine aggregate.

**Figure 4:**
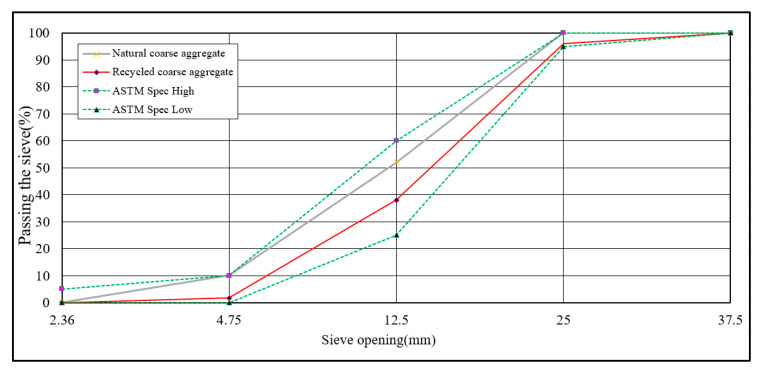
Particle size distribution curve of natural and recycled coarse aggregate.

The recycled coarse aggregate used in this study is provided by Rongmin Engineering Co., Ltd. (Keelung, Taiwan) and produced by the equipment of the Luodong sand and gravel plant (Yilan, Taiwan). Due to the current regulations of the sand and gravel plant, only natural river bed soil and stone can be processed and construction waste can not be used for processing. This study made concrete blocks in the sand and gravel plant, and made recycled aggregate after 56 days of curing, including specific gravity, water absorption, and maximum diameter, as shown in Table 9 and Table 10. The appearance of recycled coarse and fine materials is shown in Figure 2. The particle size distribution curve of natural and recycled fine aggregate is shown in Figure 3. The particle size distribution curve of natural and recycled coarse aggregate is shown in Figure 4.

### 2.2. Mix Design and Test Methods

This study explored the performance of the ICM on a GC substrate. Table 11 shows relevant parameters.

The ACI-211 mix design method was adopted for designing the concrete base material mix, as shown in Table 12. At least three specimens were prepared for each test, and relevant tests were conducted at 7, 28, and 56 days.

The important factors in the selection of the GC and ICM include the replacing cement, replacing coarse-grained material, replacing fine-grained material, coating type, coating material proportion, coating layer number, and coating material age. A water:cement ratio of 0.6 was used to test the physical properties, permeability, and microstructure of the concrete substrate. The effectiveness of GC coated with an ICM was discussed, and comparisons with a control group were performed. Table 13 shows the test items and referenced standards. Three specimens of each mixture are required for each test in this study.

## 3. Results and Discussion

### 3.1. Compressive Strength

**Figure 5:**
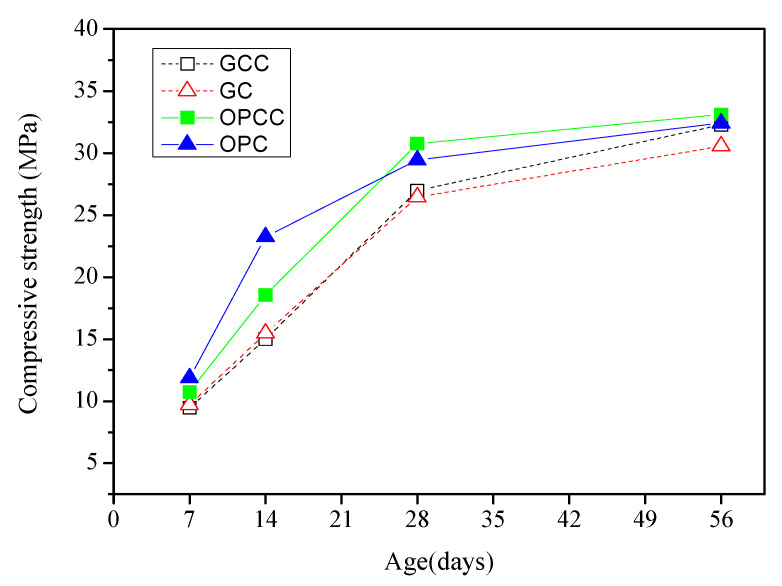
Compressive strength development curves.

Tests were conducted according to ASTM C39M-12 [49]. The universal material testing machine used in this study is made by Shimadzu Corporation. It is controlled by the hydraulic system and can be used to test the compressive and flexural properties of concrete. The maximum allowable load of the instrument is 1000 kni, and the minimum reading value is 50 kg. The speed and data are automatically controlled by computer software. Figure 5 shows the development trend of the compressive strength of GC and OPC as well as GC and OPC with an ICM (GCC and OPCC, respectively) at 7, 14, 28, and 56 days. The detailed data of the compression strength are shown in Table 14.

The compressive strength development curves of OPC and GC at 7, 14, 28, and 56 days show that the strength of GC was 2–8 MPa lower than that of OPC at each age. The compressive strength of recycled coarse and fine particles and photoelectric glass powder may be lower because of incomplete hydration that results from replacing general aggregates or cement. However, the compressive strength of GC at 56 days was only 6% lower than that of OPC. The hydration of GC tends to become more complete with age. The compressive strengths of OPCC and GCC are higher than those of OPC and GC, respectively, at 28 and 56 days. It is speculated that the ICM blocks the pores owing to a chemical reaction with the concrete substrate, resulting in a slight increase in compressive strength. The compressive strength of GCC at 56 days was slightly higher than that of GC, indicating that the ICM has a certain protective effect. In a base material with large pores, it is suitable to plug these pores to improve the concrete quality.

### 3.2. Absorption Test and Initial Surface Absorption Test

**Figure 6:**
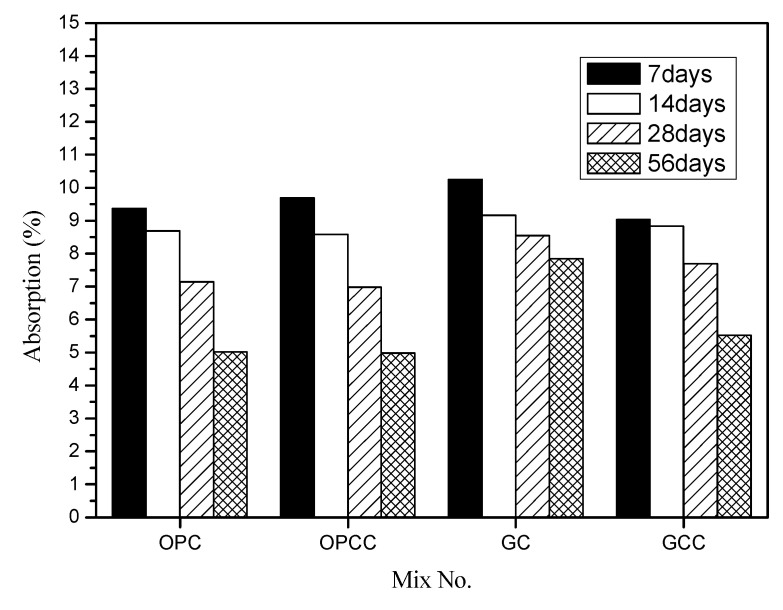
Comparison of absorption of concrete.

An absorption test was performed according to ASTM C642-13 [50]. Further, an initial surface absorption test was performed according to BS 1881-208 [56]. The compactness of pores in the concrete substrate and the protective effect of the ICM were evaluated. Figure 6 shows the absorption tests of GC and GCC as well as OPC and OPCC at 7, 14, 28, and 56 days.

The results show that the absorption rate of all groups decreased with time. The hydration reaction in the sample tended to become more complete with time, making the pores more compact [7]. The absorption rate of GC was higher than that of OPC. The compactness of recycled coarse and fine particles was lower than that of natural fine particles, leading to high absorption and high porosity. The high compactness should be caused by the old paste being attached to the recycled particles. For GC, the decrease in absorption rate with age was not obvious. Further, the absorption rates of OPCC and GCC are lower than those of OPC and GC, respectively, at 14, 28, and 56 days. It is speculated that because GCC has a high porosity, it is easier for it to penetrate the pores of the base material to block its connectivity or reduce the pore size, indicating that the ICM has a certain protective effect.

**Figure 7:**
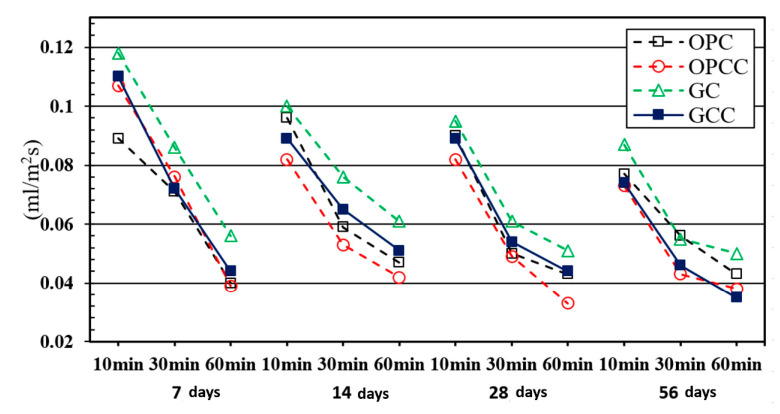
Comparison of the absorption rate of concrete.

When external water enters the specimen, it must penetrate the surface and internal capillary pores. When the pores are small or have poor connectivity, the absorption rate of the sample decrease with age. Table 15 and Figure 7 show the initial surface absorption tests of GC and GCC as well as OPC and OPCC at 7, 14, 28, and 56 days.

The experimental results showed that the absorption rate of each group decreased with age. In this experiment, GC had a high absorption rate owing to the high porosity of the recycled coarse and fine particles. However, GCC had a lower absorption rate, indicating that the ICM could adequately block the interior of the substrate. Because the ICM was easy to penetrate into the pores, resulting in obvious blocking effect, effectively reducing the water absorption [31,32,57,58].

### 3.3. Four-Pole Resistance Test

**Figure 8:**
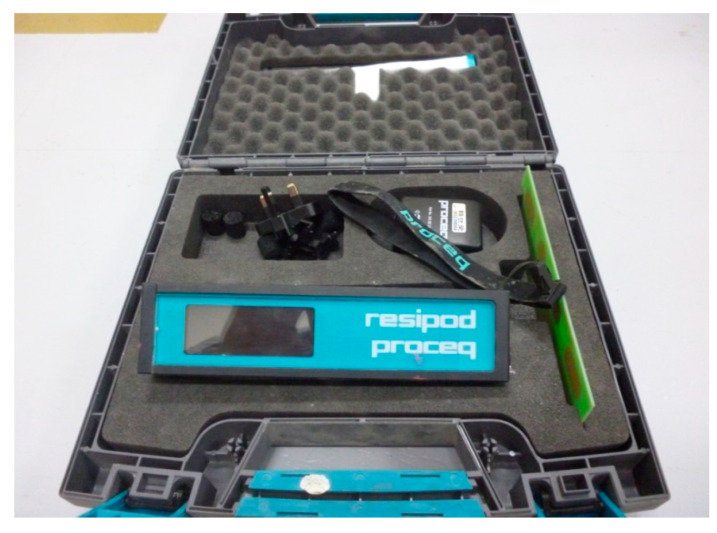
Four-pole resistance measuring instrument.

**Figure 9:**
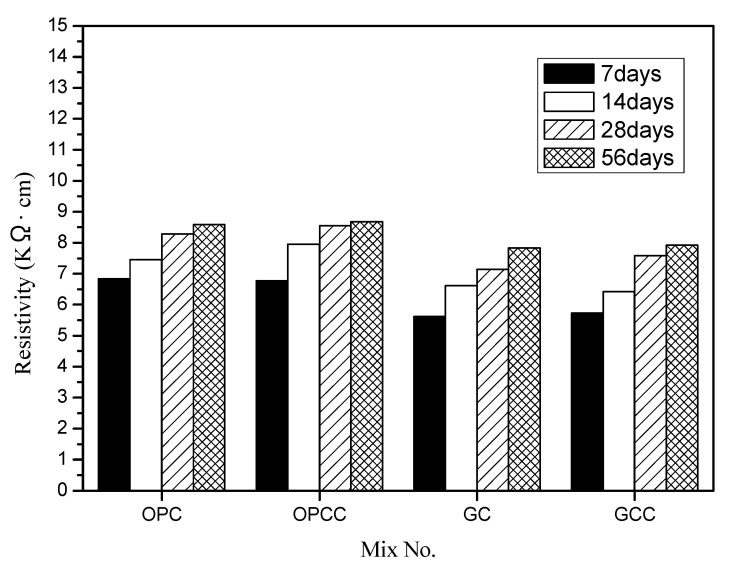
Comparison of concrete resistivity.

In this study, the concrete quadrupole resistance meter manufactured by Swiss proceq company was used to test. The resistance of the equipment is 10 mΩ, the rated current is 180 µA, the frequency is 72 Hz, the measurement range is 0~99 KΩ cm, and the accuracy is ± 1 Ω cm. The appearance of the instrument is shown in Figure 8, which conforms to AASHTO standard (38 mm probe distance). Figure 9 shows the development trend of the resistivity values of GC and GCC as well as OPC and OPCC at 7, 14, 28, and 56 days.

When the resistivity value becomes high, it can be inferred that the concrete substrate is relatively dense and the conductive path is blocked. Then, the permeability of the base material is evaluated. The results show that GC has lower resistivity than OPC at all ages. However, GCC shows increased resistivity. OPC and OPCC had the same trend. ICM can prevent the resistance from penetrating through the pores of the substrate, it caused that the OPCC and GCC specimens were shown increased resistivity.

### 3.4. Accelerated Chloride Migration Test

**Figure 10:**
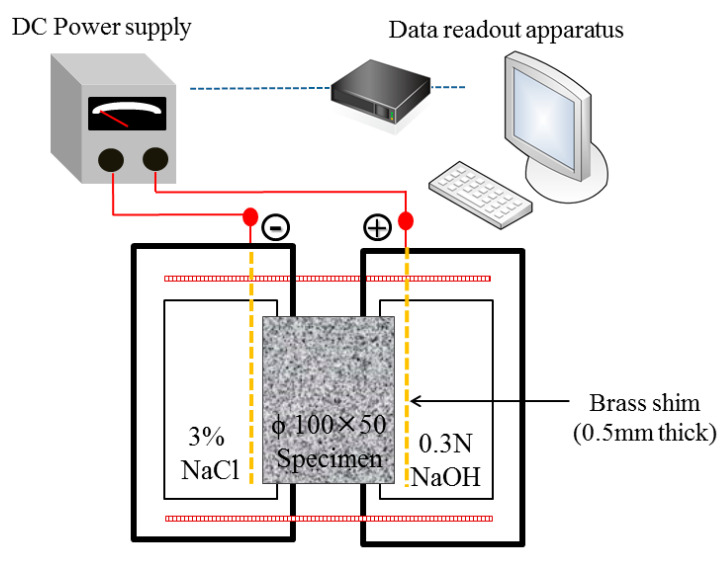
Schematic diagram of accelerated chloride migration test.

**Figure 11:**
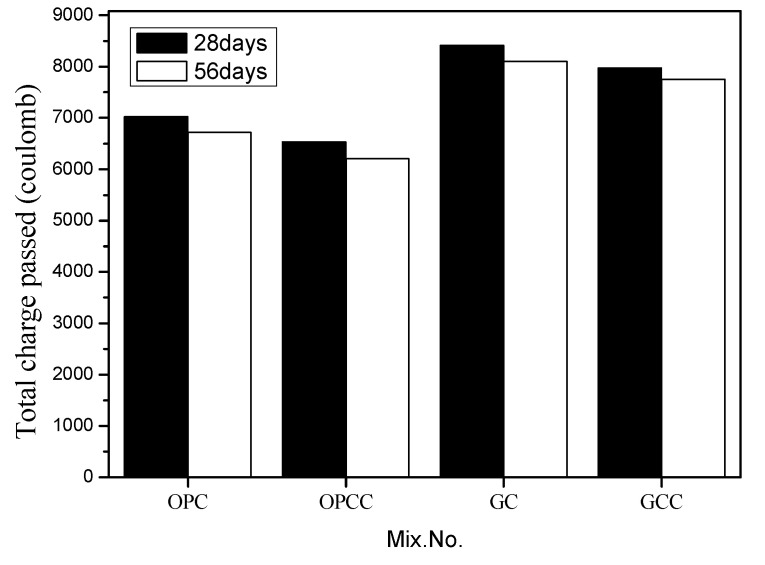
Comparison of cumulative total power supply.

An accelerated chloride migration test was conducted according to ASTM C1202-12 [52]. The test equipment is divided into two parts: vacuum equipment and current measurement equipment. The vacuum equipment includes a vacuum pump, a vacuum gauge, and a vacuum tank. The current measuring equipment includes Rapid Chloride Permeability Test (RCPT) cell, digital galvanometer, and DC power supply, as shown in Figure 10. The voltmeter is connected by a circuit, and the applied voltage is 60 V to measure its current value, the current measurement range is 0.1~1000 mA, and the voltage measurement range is 0.1~100 V. The current value of concrete was measured, and the total electricity passing through within 6 h was obtained to evaluate the ability of concrete to resist chloride ion penetration. When the total charge is lower, chloride ion penetration is judged to be low. By contrast, a high total charge indicates high penetration of chloride ions and a relatively low densification path. Figure 11 shows the accelerated chloride penetration values of GC and GCC as well as OPC and OPCC at 28 and 56 days.

The test results show that OPC has lower chloride ion penetration than GC at 28 and 56 days. When recycled coarse and fine particles and photoelectric glass powder are used to replace general aggregates or cement, incomplete hydration causes low compactness of the substrate, resulting in high chloride ion penetration [59]. However, an ICM can block external chloride ion penetration and substrate pores. OPCC and GCC had lower chloride ion penetration than OPC and GC, respectively. The infiltration also had a blocking effect.

### 3.5. Mercury Intrusion Porosimetry

A mercury intrusion porosimetry (MIP) test was conducted according to ASTM D4404-10 [53]. The mercury porosimeter in this study uses autopore IV 9500 produced by micromeritics company. The pore volume, pore distribution, porosity, and compactness can be obtained by the conversion of injected mercury. The pore measurement range can reach 0.003~360 μM. The test can be divided into two parts: high pressure and low pressure. During the low pressure analysis, the system will be pressurized to 30 psi, and then put into the high pressure tank for high pressure analysis. The maximum pressure can be pressurized to 60,000 psi. MIP can be used to determine the pore structure of the base material. The pore volume of a cement-based material can be calculated from the residual mercury volume after being pressed into the test body. In this study, porosity was defined for a 10 nm reference value. The colloidal pore structure was defined as a pore diameter less than 10 nm, and the capillary pore structure was defined as a pore diameter greater than 10 nm. The total pore content is equal to the sum of the capillary and colloidal pores.

**Figure 12:**
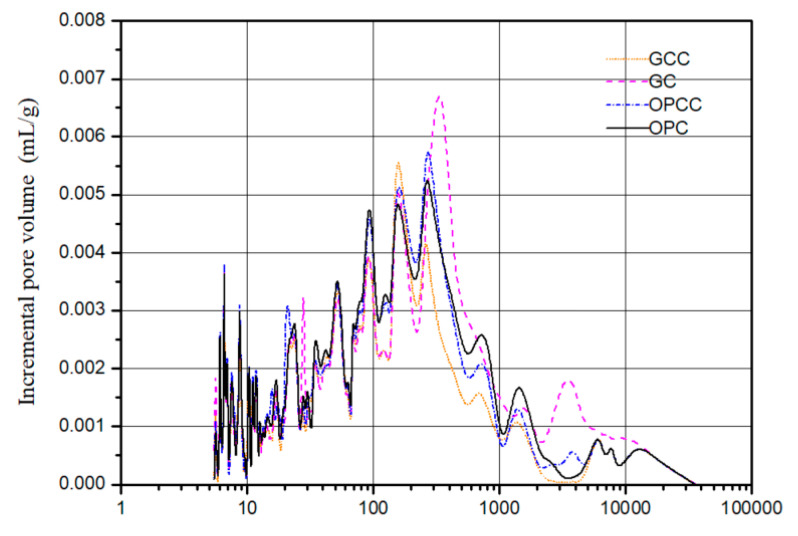
Cumulative volume diagram of capillary and colloidal pores.

**Figure 13:**
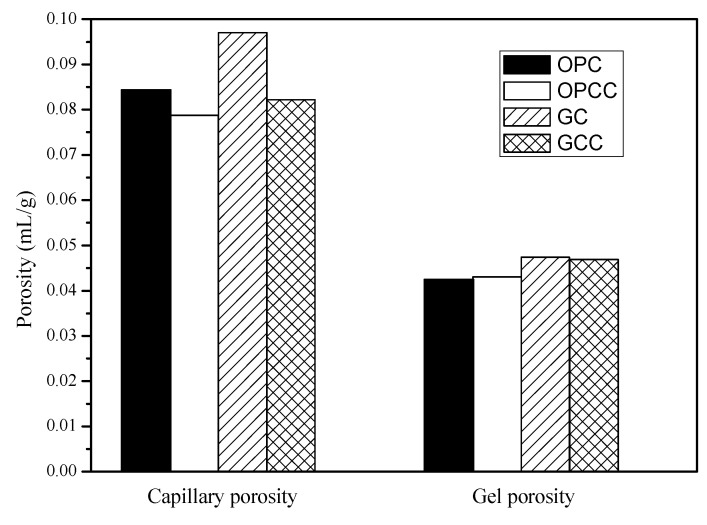
Pore distribution.

In this study, an MIP was performed at 28 days to investigate the effects of GC and ICM on the pore structure of the substrate. Figure 6 and Figure 7 show the measurement results of the capillary and colloidal pore volumes of the substrate. The results show that the porosity of GC was higher than that of OPC owing to the use of recycled particles. However, OPCC and GCC had decreased porosity. In Figure 12, the pore distribution changed markedly from 100 to 10,000 nm, and GCC had a better effect. It is speculated that owing to the large intrinsic pores in GCC, it is easy for it to penetrate the pores and produce an obvious blocking effect. As shown in Figure 13, the decrease of capillary pores with pore size greater than 10 nm is more obvious; because of its small pore size, harmful substances may not be able to penetrate the colloidal pores.

### 3.6. Scanning Electron Microscopy

**Figure 14:**
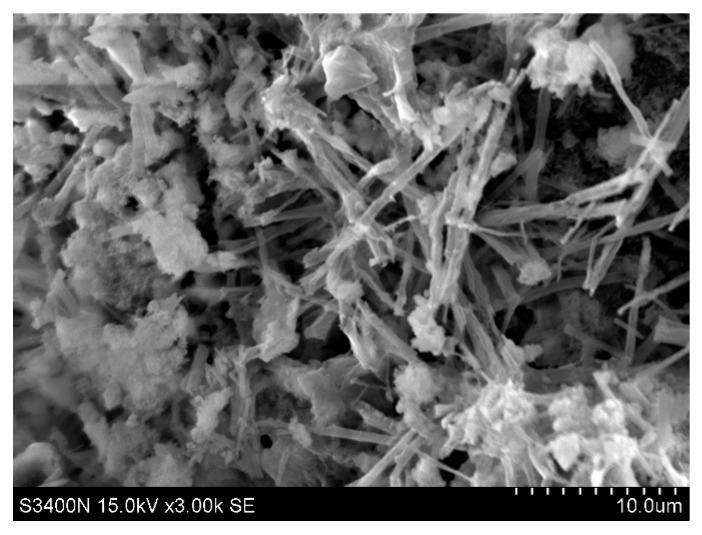
Microstructure 5 mm below the inner coating of the substrate (3000×).

**Figure 15:**
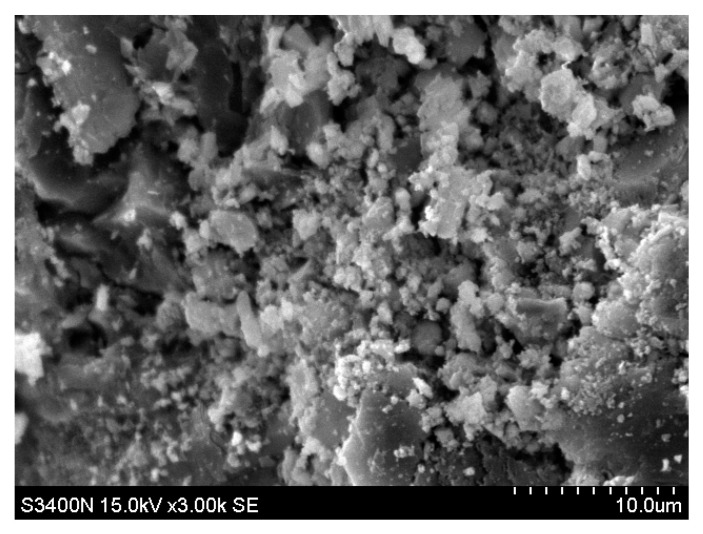
Microstructure 20 mm below the inner coating of the substrate (3000×).

The scanning electron microscope s-4800 made by Hitachi company of Japan (Hitachi, Tokyo, Japan) was used to observe the microstructure. The maximum magnification of the instrument can reach 10,0000 times. The principle of the instrument is to use the electron gun to emit high-energy electron light to impact the specimen, then use the bias signal collector to detect the excited signal, and transmit it to the cathode mapping tube through the signal amplifier. As shown in Figure 14, SEM observations revealed needle-like structures 5 mm below the substrate surface. These are speculated to be produced by the chemical reaction between the ICM and the substrate. XRD composition analysis suggests that the needle-like substance should comprise C-S-H colloid or calcium carbonate. As shown in Figure 15, no needle-like structures were observed 20 mm below the coating layer, suggesting that the needle-like structures decrease with depth.

### 3.7. XRD Spectral Analysis

**Figure 16:**
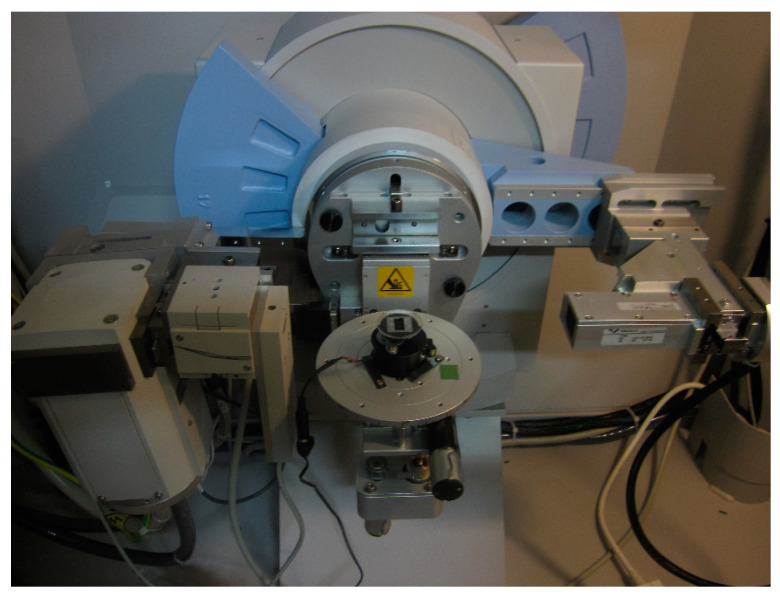
X-ray diffraction analyzer.

**Figure 17:**
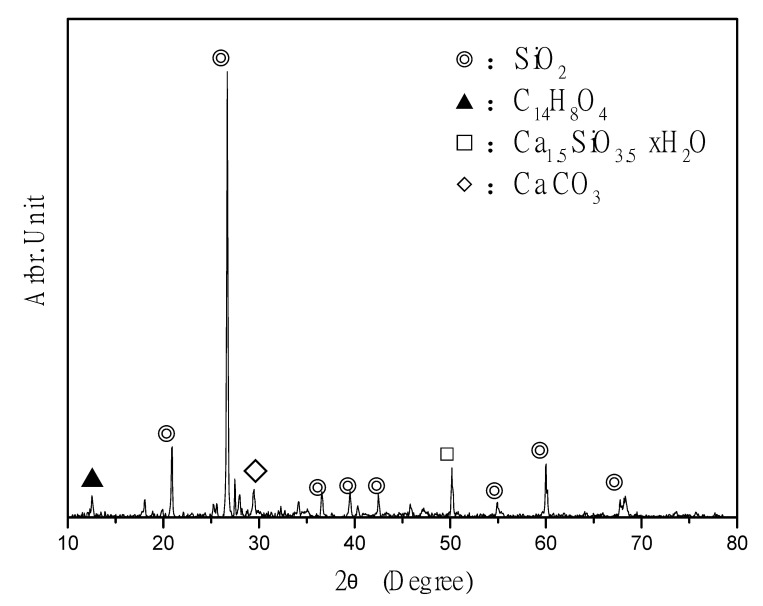
Chemical composition of ICM 5 mm below the coating layer, as determined through XRD.

**Figure 18:**
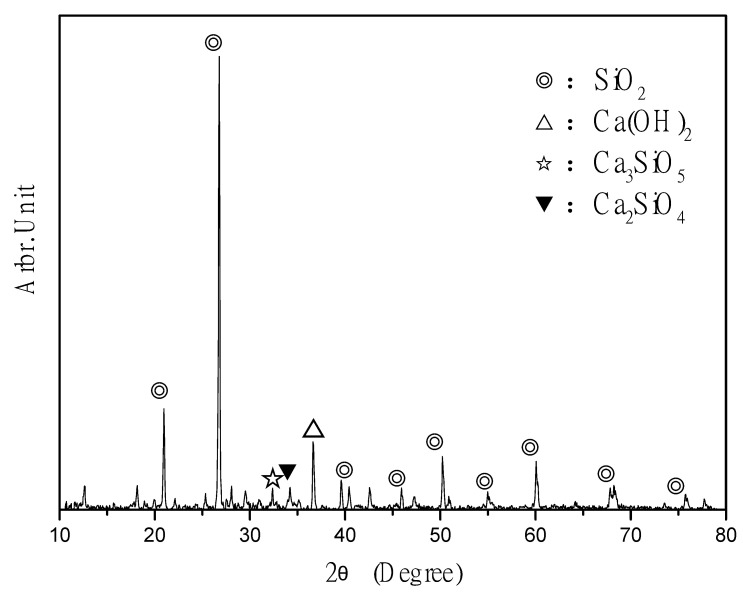
Checmical composition of pure cement substrate 20 mm below the coating, as determined through XRD.

The X-ray diffraction analyzer used in this study is panalytical x’pert Pro MPD. The principle of X-ray diffraction analysis is to irradiate the sample with monochromatic X-ray, and the diffracted X-ray will radiate along the conical surface at an angle of 2θ with the incident angle. By recording the diffraction intensity at different angles, the 2θ intensity diagram can be drawn. The appearance of the instrument is shown in Figure 16. The ICM has been proven to have a protective effect. Specifically, the ICM components penetrate the base material and produce needle-like substances in the pores that improve the compactness and protect the concrete [60,61]. Therefore, an XRD spectral analysis was performed 5 and 20 mm below the coating layer (Figure 17 and Figure 18, respectively) to clarify the chemical composition of the samples.

Compared with Figure 18, the cement-based compounds in Figure 17 are mainly silica, calcium hydroxide, C_3_S, and C_2_S. It can be inferred that the ICM does not penetrate 20 mm below the coating layer to cause a reaction. It is speculated that if the coating material does not penetrate the sealing layer to a depth of 5 mm, crystallization occurs under the sealing layer owing to the different chemical compositions. It can be inferred that ICMs can react to form crystals by penetrating the concrete, thereby improving the protective efficiency of the substrate.

## 4. Conclusions

The physical properties of GC coated with an ICM show that when recycled coarse and fine particles and photoelectric glass powder are used to replace ordinary particles or cement, the porosity may become higher owing to incomplete hydration, resulting in lower compressive strength and a higher absorption value. However, when the ICM is coated on the base material, the performance of the base material improves slightly. It is speculated that the ICM reduces the porosity of the base material to improve the quality of concrete.

Permeability tests show that GC had lower permeability than OPC at 28 and 56 days, indicating that the low compactness of the GC substrate resulted in high chloride penetration. However, an ICM can block chloride ion penetration and block the pores of the substrate.

SEM revealed that the microstructure of GCC had needle-like structures 5 mm below the substrate surface but not 20 mm below the substrate surface. It is speculated that the ICMs are permeable and produced a chemical composition with the substrate, resulting in the obvious presence of needle-like structures 5 mm below the coating layer.

## Figures and Tables

**Table 1:** Chemical constituents of solar photovoltaic glass powder.

Composition	Percentage (wt.%)
Silicon dioxide (SiO_2_)	75.92
Sodium oxide (Na_2_O)	8.84
Calcium oxide (CaO)	6.06
Ferric oxide (Fe_2_O_3_)	0.29
Aluminum oxide (Al_2_O_3_)	0.11
Magnesium oxide (MgO)	2.74
Sulfur trioxide (SO_3_)	2.3
Potassium oxide (K_2_O)	—

**Table 2:** Chemical constituents of ICM (Inorganic coating material).

Composition	Percentage (wt.%)
Silicon dioxide (SiO_2_)	15.4
Sodium oxide (Na_2_O)	3.33
Calcium oxide (CaO)	64.8
Ferric oxide (Fe_2_O_3_)	3.74
Aluminum oxide (Al_2_O_3_)	3.26
Magnesium oxide (MgO)	6.51
Sulfur trioxide (SO_3_)	2.16
Potassium oxide (K_2_O)	0.25
other	0.55

**Table 3:** Physical properties of natural fine aggregate.

Test Type	Value	Referenced Standards
fineness modulus (F.M.)	2.8	ASTM C33 [46]
the specific gravity (SSD)	2.56	ASTM C128 [47]
the water absorption (%)	1.85	ASTM C128 [47]

**Table 4:** Gradation distribution of natural fine aggregate.

Sieve Number	Mesh Size (mm)	Percentage of Stay (%)	Cumulative Percentage (%)
#4	4.75	0.4	0.4
#8	2.38	16.8	17.2
#16	1.18	20.5	37.7
#30	0.60	20.2	57.9
#50	0.30	18.4	76.3
#100	0.15	13.5	89.8
chassis	—	10.2	—

**Table 5:** Physical properties of natural coarse aggregate.

Test Type	Value	Referenced Standards
the specific gravity (SSD)	2.53	ASTM C127 [48]
the water absorption (%)	1.68	ASTM C127 [48]
maximum diameter (mm)	19.05	ASTM C127 [48]

**Table 6:** Gradation distribution of natural coarse aggregate.

Sieve Number	Mesh Size (mm)	Percentage of Stay (%)	Cumulative Percentage (%)
1 1/2	37.5	0	0
1	25	0	0
1/2	12.5	47.98	47.98
#4	4.75	41.94	89.92
#8	2.38	10.08	100

**Table 7:** Physical properties of recycled fine aggregate.

Test Type	Value	Referenced Standards
the specific gravity (SSD)	2.42	ASTM C128 [46]
the water absorption (%)	6.16	ASTM C128 [46]
fineness modulus (F.M.)	2.43	ASTM C33 [47]

**Table 8:** Gradation distribution of recycled fine aggregate.

Sieve Number	Mesh Size (mm)	Percentage of Stay (%)	Cumulative Percentage (%)
3/8”	9.53	0.0	0.0
#4	4.75	0.0	0.0
#8	2.38	1.8	1.8
#16	1.18	26.6	28.4
#30	0.60	23.8	52.2
#50	0.30	20.8	73.0
#100	0.15	15.0	88.0
#200	0.075	12.0	100.0

**Table 9:** Physical properties of recycled coarse aggregate.

Test Type	Value	Referenced Standards
the specific gravity (SSD)	2.47	ASTM C127 [48]
the water absorption (%)	4.88	ASTM C127 [48]
maximum diameter (mm)	19.00	ASTM C127 [48]

**Table 10:** Gradation distribution of recycled coarse aggregate.

Sieve Number	Mesh Size (mm)	Percentage of Stay (%)	Cumulative Percentage (%)
1 1/2	37.5	0	0
1	25	4	4
1/2	12.5	58	62
#4	4.75	36.26	98.26
#8	2.38	1.74	100

**Table 11:** Concrete test variables.

Type	Control Group (OPC)	Green Concrete (GC)	Coating (Control Group) (OPCC)	Green Concrete (Including Coating) (GCC)
Replace cement	—	Photoelectric glass powder 5%	—	Photoelectric glass powder 5%
Replace coarse aggregate	—	Recycled coarse aggregate 20%	—	Recycled coarse aggregate 20%
Replace fine aggregate	—	Recycled fine aggregate 15%	—	Recycled fine aggregate 15%
Paint type	—	—	ICM1	ICM1
Coating material proportion	—	—	5:2	5:2
Number of coating layers	—	—	2 layers	2 layers
Age of coating materials	—	—	Day 1 coating	Day 1 coating

**Table 12:** Concrete mix design (kg/m^3^).

Mix No.	w/c	Water	Cement	Solar Photovoltaic Glass Powder	Coarse Aggregates	Fine Aggregates	Replace Cement	Replace Coarse Aggregate	Replace Fine Aggregate
**OPC**	0.6	240	396	0	1096	890	0	0	0
**OPCC**	0.6	240	396	0	1096	890	0	0	0
**GC**	0.6	240	376	18.8	876	756	20	220	134
**GCC**	0.6	240	376	18.8	876	756	20	220	134

**Table 13:** Test items and referenced standards.

Test Type	Test Method	Specimen Dimensions(cm) and Types	Referenced Standards
Mechanical properties	Compressive strength test	ϕ 10×20 (concrete)	ASTM C39m-12 [49]
Permeability	Absorption test	ϕ 10×5 (concrete)	ASTM C642-13 [50]
	Four pole resistance test	ϕ 10×20 (concrete)	ASTM C876 [51]
	Accelerated chloride migration test	ϕ 10×5 (concrete)	ASTM C1202-12 [52]
Characterization	Mercury intrusion porosimetry	1×1×1 (concrete)	ASTM D4404-10 [53]
	Scanning electron microscope	1×1×1 (mortar)	ASTM C1723 [54]
	XRD spectrum analysis	Powders	ASTM C1365 [55]

**Table 14:** The detailed data of the compression strength.

Mix No.	7 Day	Standard Deviations	14 Day	Standard Deviations	28 Day	Standard Deviations	56 Day	Standard Deviations
**OPC**	11.87	1.86	23.25	1.56	29.45	1.94	32.41	2.20
**OPCC**	10.76	2.05	18.56	1.62	30.77	1.95	33.12	2.15
**GC**	9.71	2.18	15.48	1.99	26.46	2.72	30.57	1.98
**GCC**	9.48	1.96	15.02	2.31	26.98	1.78	32.28	1.87

**Table 15:** Initial surface absorption of concrete (ml/m^2^s).

Mix No.	7 Day			14 Day			28 Day			56 Day		
	10 min	30 min	60 min	10 min	30 min	60 min	10 min	30 min	60 min	10 min	30 min	60 min
OPC	0.089	0.071	0.04	0.096	0.059	0.047	0.09	0.05	0.043	0.077	0.056	0.043
OPCC	0.107	0.076	0.039	0.082	0.053	0.042	0.082	0.049	0.033	0.073	0.043	0.038
GC	0.118	0.086	0.056	0.1	0.076	0.061	0.095	0.061	0.051	0.087	0.055	0.05
GCC	0.11	0.072	0.044	0.089	0.065	0.051	0.089	0.054	0.044	0.074	0.046	0.035

## Data Availability

Data sharing is not applicable to this article.
